# Reviewers

**DOI:** 10.1111/jdi.12429

**Published:** 2015-10-27

The publication of invaluable papers in the *Journal of Diabetes Investigation* depends on the prompt, careful review of submitted manuscripts. We would like to thank the Editorial Board members, the International Editorial Board members, the Assistant Editorial Board members and the following experts for reviewing manuscripts from September 1, 2014 to August 31, 2015.

Atsuko Abiko

Norio Abiru

Toru Aizawa

Kamel Ajlouni

William Alazawi

Hitoshi Ando

Keiko Arai

Atsushi Araki

Shin-ichi Araki

Yoshimasa Aso

Masayuki Baba

Preethi Balan

Yuqian Bao

Larry Baum

Shalini Bhaskar

Phillip J Brantley

Duane C Kissell

Guoshuai Cai

Subrata Chakrabarti

Chih-Jen Chang

Feng-Yee Chang

Tien-Jyun Chang

Yi-Cheng Chang

Cheng-Hsu Chen

Gang Chen

Harn-Shen Chen

Jin-Shuen Chen

Ching lung Cheung

Kitty Cheung

Pik To Cheung

Dong Hyeok Cho

Jae Hyoung Cho

Nam Cho

Han Seok Choi

Hyung Jin Choi

Chih-Hsun Chu

I-Hua Chu

Sung Wan Chun

Erkan Coban

Makoto Daimon

Carolyn Deacon

Takahisa Deguchi

Alexander V. Dreval

Vincent Durlach

Nabil El-Naggar

Masanori Emoto

Bo-Guang Fan

Liang Feng

Martin Finian

Chia-Po Fu

Giorgio Fuiano

Kei Fujimoto

Shimpei Fujimoto

Tomomi Fujisawa

Yoshihito Fujita

Yukihiro Fujita

Yoshio Fujitani

Michiaki Fukui

Kengo Furuichi

Abdul Ghani

Carlos J Gois

Atsushi Goto

Honglei Guo

Kyoung Hwa Ha

Yusuke Hada

Tomoya Hamaguchi

Kumiko Hamano

Akihiro Hamasaki

Seung Jin Han

Kazuo Hara

Masumi Hara

Mariko Harada-Shiba

Shin-ichi Harashima

Takeshi Hayashi

Yasuaki Hayashino

Takumi Hirata

Takahisa Hirose

Hiroshi Inoue

Yushi Hirota

Kerry Hitos

Osamu Hori

Yukio Horikawa

Masaaki Hoshiga

Mohsen Hosseini

An-Tsz Hsieh

Chang-Hsun Hsieh

Hui-Min Hsieh

Cheng Hu

Chii-Min Hwu

Jon Hyett

Atsuhiro Ichihara

Hiroshi Ikegami

Junta Imai

Fumiaki Imamura

Minako Imamura

Toyoshi Inoguchi

Kazuo Inoue

Manami Inoue

Hisamitsu Ishihara

Hideki Ishii

Naoko Iwasaki

Yasumasa Iwasaki

Masanori Iwase

Edward Janus

Jiann-Shing Jeng

Ja Young Jeon

Mihai Juncar

Hideki Kamiya

Kyuzi Kamoi

Keizo Kanasaki

Akio Kanazawa

Ippei Kanazawa

Shizuka Kaneko

Hideaki Kaneto

Naoto Katakami

Koichi Kato

Tomoyuki Katsuno

Masanobu Kawakami

Tomoyuki Kawamura

Daiji Kawanami

Eiji Kawasaki

Yoshiaki Kido

Chong Hwa Kim

Chul-Hee Kim

Hyeon Chang Kim

Jae Hyeon Kim

Min Hee Kim

Sun Hee Kim

George King

Tadahiro Kitamura

Seung-Hyun Ko

Teruhiko Koike

Mitsuhisa Komatsu

Alice Kong

Shigeo Kono

Bokyung Koo

Daisuke Koya

Hidenori Koyama

Akira Kubota

Naoto Kubota

Daitarou Kurosaka

Takeshi Kurose

Soo-Heon Kwak

Hyuk-Sang Kwon

Ting-I Lee

Alex Lee

ChangWon Lee

Chien-Hsing Lee

Eun Jig Lee

Eun Young Lee

Hae Young Lee

I-Te Lee

Ji-Hyun Lee

Lu-Li Lee

Suk Woo Lee

Won-Young Lee

Yau-Jiunn Lee

Yongho Lee

Klaus Levin

Chanjuan Li

Duo Li

Hung-Yuan Li

Qizhou Lian

Chou-Ching K. Lin

Kun-Der Lin

Shih-Yi Lin

Tsung-Hsin Lin

Wen-Yuan Lin

Giieh-Hua Lu

Kuo-Cheng Lu

Andrea Luk

Shiro Maeda

Hiroshi Maegawa

Shinya Makino

Yuichi Makino

Shoichi Maruyama

Hiroaki Masuzaki

Masafumi Matsuda

Munehide Matsuhisa

Hideyuki Matsumoto

Shinichi Matsumoto

Huang Meng-Chuan

Silvia Migliaccio

Takashi Miki

Hirofumi Misu

Jun-ichiro Miyagawa

Hideaki Miyoshi

Tetsuya Mizoue

Hiroki Mizukami

Andrea Montagnani

Aya Morimoto

Katsutara Morino

Masaaki Muramatsu

Ryoji Nagai

Shoichiro Nagasaka

Akinobu Nakamura

Nobuhisa Nakamura

Yoshio Nakata

Takao Nammo

Takuma Narita

Koji Nata

Aziz Nather

Masahiro Nishi

Yoshihiko Nishio

Mitsuhiko Noda

Hiroyuki Nodera

Takashi Nomiyama

Susumu Ogawa

Masahito Ogura

In-Hwan Oh

Ken Ohashi

Tsuyoshi Ohkura

Yosuke Okada

Yoshihisa Okamoto

Hiroaki Okazaki

Ryo Okazaki

Kenji Osafune

Haruhiko Osawa

Tsuguhito Ota

Peter Vestergaard

Jiemin Pan

Maria Panagioti

Giuseppe Papa

Cheol Whee Park

Mi Kyoung Park

Yong-Moon Park

Dee Pei

Deniz Rende

Sang Youl Rhee

Yoshifumi Saisho

Kazuhiko Sakaguchi

Naoki Sakane

Hiroshi Sakura

Takashi Sakurai

Kazunori Sango

Hideyuki Sasaki

Toshiyasu Sasaoka

Susumu Sawada

Yusuke Seino

Naoto Seki

Shaolin Shi

Kenichi Shikata

Akira Shimada

Hideaki Shimizu

Seiya Shimoda

Iichiro Shimomura

Hirohito Sone

Ki-Ho Song

Kee-Ho Song

James Sowers

Marian Stelmach

Dirk J. Stenvers

Mary C Stephenson

SC Su

Sui-Lung Su

Takayoshi Suganami

Yoshihisa Sugimura

Takashi Sugiyama

Takehiro Sugiyama

Takuya Suguyama

Prisana Suwannaporn

Atushi Suzuki

Ryou Suzuki

Kiyoshi Suzuma

Gregory Szot

Yasuharu Tabara

Yuji Tajiri

Mitsuyoshi Takahara

Masanori Takahashi

Toshinari Takamura

Yasuhiro Takeuchi

Yoshifumi Tamura

Nagaaki Tanaka

Yasushi Tanaka

Clara Tang

Junichi Tani

Matsuo Taniyama

Fuminori Tatsumi

Yasuo Terauchi

Sarah Tersey

Kazuyuki Tobe

Tatsuya Tominaga

Masao Toyoda

Satoru Tsujii

Shin Tsunekawa

Yasuko Uchigata

Hiroyuki Umegaki

Kazunori Utsunomiya

Takashi Uzu

Marleen MJ van Greevenbroek

Nataša Veličković

Jun Wada

Chao-Hung Wang

Eric Wang

Jun-Sing Wang

Jun Wang

Maria Warwas

Jong Chul Won

Chau-Chung Wu

Shenghui Wu

Kunimasa Yagi

Kentaro Yamada

Yuichiro Yamada

Kazuya Yamagata

Sho-ichi Yamagishi

Masahiro Yamamoto

Ritsuko Yamamoto-Honda

Yasuhiko Yamamoto

Toshihiko Yanase

Jichun Yang

Wei-Shiung Yang

Xilin Yang

Hitoshi Yasuda

Yohichi Yasunami

Norihide Yokoi

Koichi Yokono

Tamotsu Yokota

Koutaro Yokote

Masayasu Yoneda

Narihito Yoshioka

Pei Yu

Yukio Yuzawa

Sen Zhang

Yaqing Zhang

Jian Zhou

Faming Zhu

Volha Zhukouskaya

